# Circulating miRNAs as Non-Invasive Biomarkers in Pancreatic Cancer: A Two-Phase Plasma-Based Study

**DOI:** 10.3390/jcm14186430

**Published:** 2025-09-12

**Authors:** Vlad Alexandru Ionescu, Gina Gheorghe, Coralia Bleotu, Liliana Puiu, Cristina Mambet, Camelia Cristina Diaconu, Carmen Cristina Diaconu

**Affiliations:** 1 Faculty of Medicine, University of Medicine and Pharmacy Carol Davila Bucharest, 050474 Bucharest, Romania; vladalexandru.ionescu92@gmail.com (V.A.I.); gheorghe_gina2000@yahoo.com (G.G.); 2 Internal Medicine Department, Clinical Emergency Hospital of Bucharest, 105402 Bucharest, Romania; 3 Department of Cellular and Molecular Pathology, Stefan S. Nicolau Institute of Virology, Romanian Academy, 030304 Bucharest, Romania; puiu_liliana@yahoo.com (L.P.); cristina.mambet@gmail.com (C.M.); carmen.diaconu@virology.ro (C.C.D.); 4 Research Institute of the University of Bucharest (ICUB), University of Bucharest, 060023 Bucharest, Romania; 5 Academy of Romanian Scientists, 050085 Bucharest, Romania; 6 Hematology Department, Emergency University Clinical Hospital of Bucharest, 050098 Bucharest, Romania

**Keywords:** miRNA, real-time PCR, non-invasive biomarkers, circulating biomarkers, pancreatic cancer

## Abstract

**Background/Objectives:** MiRNAs have demonstrated promising roles in the diagnosis of pancreatic cancer and in the prognostic assessment of affected patients. **Methods:** We conducted a prospective pilot study including 23 patients diagnosed with advanced-stage pancreatic cancer and 10 healthy controls, matched by age and sex. In the screening phase, we evaluated the expression of 176 miRNAs in pooled plasma samples from both groups using real-time PCR. Subsequently, we validated the overexpression of selected miRNAs in individual plasma samples using the same technique. Statistical analysis was performed using IBM SPSS Statistics version 29. **Results:** During the screening phase, 22 miRNAs exhibited differential expression in patients with pancreatic cancer compared to healthy controls. Among these, hsa-miR-100-5p (27.8-fold increase), hsa-miR-122-5p (7.5-fold), hsa-miR-885-5p (7.2-fold), hsa-miR-34a-5p (5.7-fold), and hsa-miR-193a-5p (4.4-fold) showed the most pronounced upregulation. In the validation phase, all five candidates demonstrated significant overexpression in individual plasma samples (*p* < 0.001). Their circulating levels also showed associations with tumor stage (*p* < 0.05). **Conclusions:** Our findings highlight a distinct circulating miRNA signature associated with advanced pancreatic cancer, supporting the potential role of hsa-miR-100-5p, hsa-miR-122-5p, hsa-miR-885-5p, hsa-miR-34a-5p, and hsa-miR-193a-5p as minimally invasive biomarkers for disease detection and staging. Larger, multicenter studies including early-stage patients and disease control groups will be required to validate these biomarkers and determine their clinical utility.

## 1. Introduction

Micro ribonucleic acid (miRNAs) are small RNA molecules, consisting of 19–24 nucleotides, that play a crucial role in post-transcriptional gene regulation [1,2]. Thus, it is estimated that approximately 60% of all human genes are regulated by these molecules [1]. MiRNA was first discovered by Victor Ambros et al. in 1993 [3]. Since then, these molecules have been extensively studied, and thousands of miRNAs have been identified in both prokaryotic and eukaryotic organisms [1]. In humans, approximately 2600 miRNAs have been characterized, including their sequences, genomic locations, and transcriptional functions [1].

MiRNAs are essential for a wide range of fundamental cellular processes, including proliferation, differentiation, metabolism, apoptosis, intercellular signaling, and hematopoiesis [1]. In addition, miRNAs are critically involved in oncogenesis and are generally classified into two main categories: tumor-suppressor miRNAs and oncogenic miRNAs (oncomiRs) [1,2,3].

The aggressive nature of pancreatic malignancies and the lack of reliable diagnostic biomarkers have led to the investigation of miRNAs as promising tools for the development of diagnostic and predictive scoring systems and for improving the therapeutic management of these patients [3,4]. Among the advantages of using miRNAs are their high stability in serum and the feasibility of non-invasive detection in circulation [5,6]. Evidence from the literature supports the notion that aberrant miRNA expression can be both a cause and a consequence of oncogenesis [7]. Aberrant miRNA profiles have been associated with widespread disruption of cellular homeostasis, including uncontrolled cell proliferation, the inhibition of tumor suppressor genes, and the modulation of apoptosis and angiogenesis, as well as tumor cell invasion and metastasis [7,8]. Although many studies have focused on tissue-specific miRNA expression in pancreatic cancer, fewer data are available on circulating miRNAs [9,10,11]. This represents a critical gap, as circulating miRNAs may reflect systemic tumor biology and provide minimally invasive biomarkers for clinical use.

Despite growing interest, limited data are available regarding intercellular communication mediated by miRNAs in pancreatic cancer [1,7]. Furthermore, a single miRNA can target multiple genes, and a single gene may be regulated by multiple miRNAs, suggesting an additive effect in the progression of disease [1,7]. Moreover, the use of miRNAs as therapeutic targets in pancreatic cancer is associated with significant limitations [1,7]. One of the most critical challenges is the incomplete understanding of the potential effects and toxicity levels of chemically modified miRNAs on healthy cells [1,7]. In conclusion, pancreatic cancer remains a leading cause of cancer-related mortality worldwide, and the involvement of miRNAs in the clinical management of these patients represents a promising avenue for improving outcomes.

Considering the limited data on circulating miRNA expression in pancreatic cancer, our study aimed to characterize and compare plasma miRNA profiles between patients with pancreatic cancer and healthy individuals, in order to identify differentially expressed miRNAs with potential diagnostic or prognostic value. To achieve this, we employed a two-phase plasma-based approach, consisting of an initial screening phase followed by validation in individual samples, to strengthen the robustness of our findings. The pivotal role of miRNAs in key biological processes—such as cell survival, apoptosis, proliferation, invasion, metastasis, and drug response—highlights their potential as diagnostic, prognostic, and therapeutic targets. The restoration of tumor-suppressive miRNA levels and inhibition of oncogenic miRNAs in healthy tissues may help maintain endogenous anti-tumor regulatory mechanisms.

## 2. Materials and Methods

**Study Design**

We conducted an exploratory pilot study that included 23 patients diagnosed with stage III or IV pancreatic cancer (Group 1) and 10 healthy controls (Group 2), matched for age and sex distribution. Patients were eligible if they had histologically confirmed pancreatic cancer. Exclusion criteria included prior malignancy within the last five years, chronic liver disease with decompensation, acute infections at sampling, or recent systemic therapy. Controls were adult volunteers with no history of cancer, pancreatic disease, or biliary obstruction, and normal routine biochemistry. 

**Ethical Approval**

The study was approved by the Ethics Committee of the Clinical Emergency Hospital Bucharest (Approval No. 3929/12.04.2021). In addition, a research collaboration framework agreement was established between the Clinical Emergency Hospital Bucharest and the “Stefan S. Nicolau” Institute of Virology (Contract No. 560/12.04.2022), allowing for the processing of biological samples at the Institute of Virology. Written informed consent was obtained from each participant prior to inclusion in the study, authorizing the collection of blood samples and the use of personal data for genomic analysis.

**Sample Collection and Plasma Isolation**

We collected 6 mL of peripheral blood from each participant in EDTA tubes and transported it within two hours to the “Stefan S. Nicolau” Institute of Virology, Bucharest. In the initial processing stage, samples were centrifuged and plasma was isolated and aliquoted into Eppendorf tubes, which were stored at −80 °C until further analysis, following standard pre-analytical handling recommendations.

**MiRNA Profiling and Validation**

In the first phase, screening for 176 miRNAs was performed using pooled plasma samples from pancreatic cancer patients and healthy controls, employing real-time polymerase chain reaction (PCR) technology. Pooling was chosen to reduce inter-individual variability and to increase the likelihood of detecting robust and consistent miRNA dysregulation. To minimize potential bias, equal plasma volumes from each participant were included in the pooled sample. Importantly, the miRNAs that demonstrated the most pronounced differential expression between pancreatic cancer patients and controls during the pooled screening were subsequently re-assessed and validated in individual plasma samples, in order to confirm reproducibility and reduce the risk of artifacts introduced by pooling.

Total miRNA was extracted from plasma samples using the miRNeasy Serum/Plasma Advanced Kit (Qiagen, Cat. No. 217204), following the manufacturer’s instructions. To monitor RNA isolation efficiency and normalize for technical variation, a spike-in RNA control was added using the miRCURY RNA Spike-In Kit for RT (Qiagen, Cat. No. 339390). Complementary DNA (cDNA) synthesis was subsequently performed using the miRCURY LNA RT Kit (Qiagen, Cat. No. 339340), according to the protocol provided by the manufacturer. Quantitative analysis of miRNA expression was performed using the miRCURY LNA miRNA Focus PCR Panel for Serum/Plasma (Qiagen, Cat. No. 339325/YAHS106YC-8), which includes a panel of pre-selected circulating miRNAs relevant to cancer. Amplification was carried out using the miRCURY LNA SYBR Green PCR Kit (Qiagen, Cat. No. 339346), in accordance with the manufacturer’s protocol. PCR reactions were conducted under standardized cycling conditions, and fluorescence data were collected at the end of each amplification cycle. Normalization was performed using both exogenous spike-in RNA controls (miRCURY RNA Spike-In Kit, Qiagen) to correct for technical variation, and endogenous reference miRNAs provided in the Qiagen panel, specifically hsa-miR-16-5p and hsa-miR-191-5p. These endogenous controls were stably expressed across samples and used as internal references for all target miRNAs, in accordance with the recommendations of the Minimum Information for Publication of Quantitative Real-Time PCR Experiments (MIQE). Hemolysis was assessed and no samples were excluded for this reason. All reactions were run in duplicate to ensure reproducibility.

**Statistical Analysis**

Data were entered into a Microsoft Excel database and subsequently analyzed using IBM SPSS Statistics version 29. Descriptive statistics were used to characterize the study population in terms of demographics, comorbidities, and laboratory parameters. Continuous variables are presented as the mean ± standard deviation (SD), minimum and maximum values, and interquartile ranges (25th, 50th, and 75th percentiles). To assess correlations between continuous biological variables (e.g., liver function tests), a Spearman rank correlation matrix was computed and graphically represented using a heatmap format.

For the screening phase of circulating miRNAs, relative expression values were calculated using the 2^−ΔΔCt^ method, with the healthy pooled sample serving as the calibrator (normalized to 1). In the validation phase, individual miRNA expression levels were compared using the Mann–Whitney U test, given the small sample size and potential deviations from normality. To account for multiple comparisons across the five validated miRNAs, *p*-values were further adjusted using the false discovery rate (FDR) correction according to the Benjamini–Hochberg method, and both unadjusted and adjusted *p*-values are reported.

Finally, to identify clinical or paraclinical parameters potentially associated with plasma miRNA expression, simple linear regression analyses were conducted. Each miRNA was treated as the dependent variable, while tumor stage, smoking status, alcohol consumption, diabetes mellitus, and history of acute or chronic pancreatitis were included as independent predictors. The results were reported as confidence intervals (95% CI) and corresponding *p*-values. Variables with *p* < 0.05 were considered statistically significant.

## 3. Results

### 3.1. Demographic and Clinical Characteristics of Pancreatic Cancer Patients

**Figure 1:**
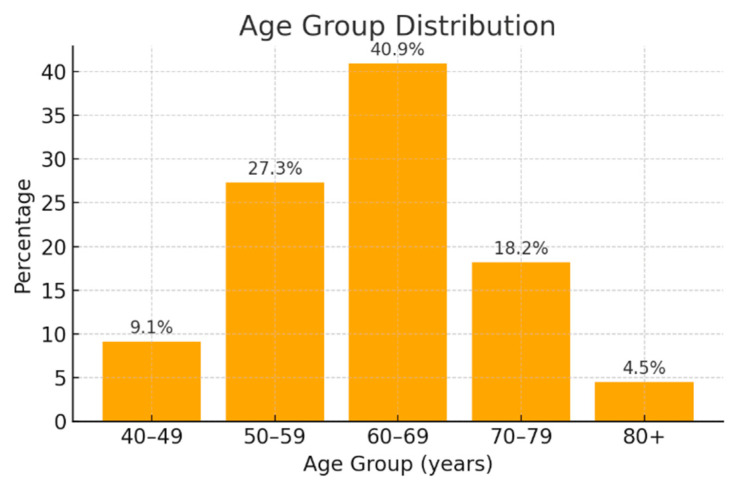
Age group distribution of the patients.

In the initial phase of the study, a descriptive analysis of the cohort was performed. A predominance of female patients was observed (68.2% female versus 31.8% male), along with a higher frequency of individuals aged 60–69 years (Figure 1). Additionally, the proportion of patients under the age of 50 was low, accounting for only 9.1% of the study population.

**Figure 2:**
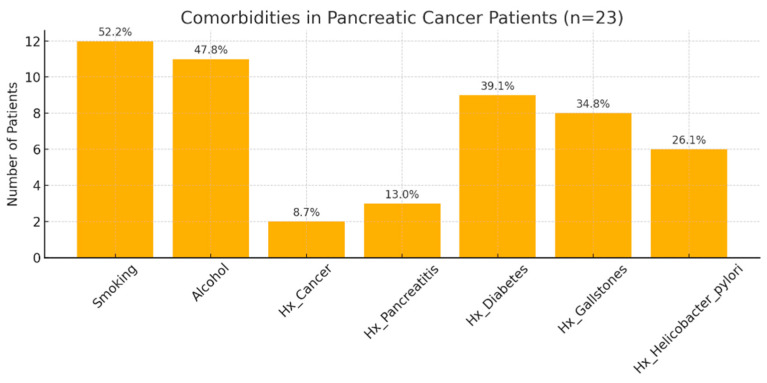
Comorbidities of pancreatic cancer patients included in the study (n = 23). Hx = reported medical history. Values are expressed as percentages of the total cohort.

Comorbidity analysis revealed a high prevalence of diabetes mellitus (39.1%), followed by gallstone disease (34.8%) and a history of acute or chronic pancreatitis (13%) (Figure 2). Alcohol consumption was reported by 47.8% of patients, whereas 52.2% were current or former smokers. In addition, 26.1% of patients had a documented history of Helicobacter pylori infection, and 8.7% reported previous malignancies—one with colorectal cancer and another with cutaneous squamous cell carcinoma, both diagnosed more than five years before the current pancreatic cancer diagnosis (Figure 2).

### 3.2. Biochemical Alterations and Tumor Pathological Features

**Figure 3:**
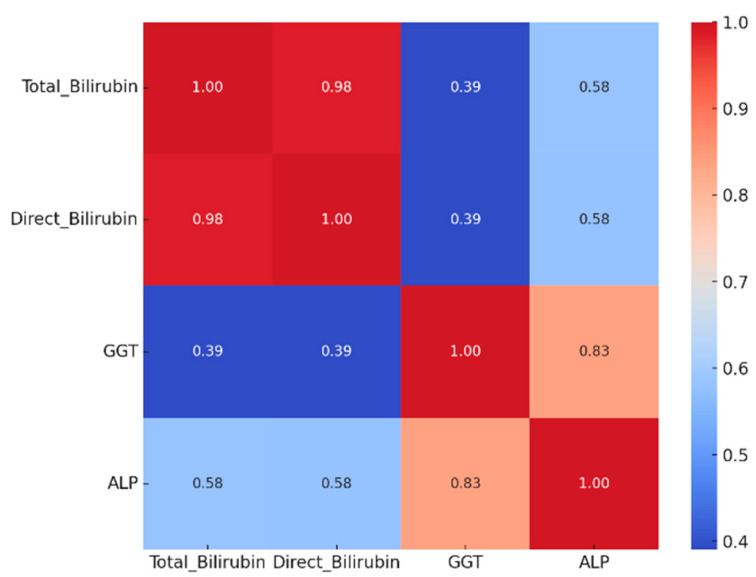
Spearman correlation heatmap showing the strength and direction of associations between cholestatic markers (Total bilirubin, Direct bilirubin, GGT, and ALP). Values inside the squares represent the Spearman correlation coefficients (r). Color intensity indicates the correlation magnitude (from blue = weak to red = strong). All correlations were tested for statistical significance using Spearman’s rank correlation.

We further analyzed the biological profile of the patients included in the study. The mean hemoglobin level was 12.11 g/dL, with 25% of patients exhibiting values below 11.25 g/dL, suggesting mild to moderate anemia in a substantial proportion of the cohort (Table 1). The mean white blood cell count was 7985.65/mm^3^, and the mean platelet count was 263,696/mm^3^, with only a small proportion of patients presenting with leukocytosis or thrombocytosis (Table 1). Approximately 25% of patients had serum glucose levels exceeding 126.5 mg/dL, consistent with the high prevalence of diabetes mellitus in the study population. Hepatic transaminase levels demonstrated marked variability, with mean values of 218.13 U/L for aspartate aminotransferase (AST) and 284.22 U/L for alanine aminotransferase (ALT), indicating hepatocellular injury in approximately half of the patients (Table 1). Markers of cholestasis were significantly elevated, reflecting biliary obstruction or advanced disease (Table 1). To further explore the potential interrelationships among these markers, a Spearman correlation matrix was constructed (Figure 3), revealing strong positive correlations between total bilirubin, direct bilirubin, alkaline phosphatase, and gamma-glutamyl transferase (GGT), suggesting a common pathophysiological substrate related to cholestasis severity. Furthermore, we observed a systemic inflammatory response, with mean C-reactive protein (CRP) levels of 30.67 mg/L and a mean erythrocyte sedimentation rate (ESR) of 35.8 mm/h (Table 1). Other parameters, such as cholesterol, triglycerides, and uric acid, were within normal or near-normal ranges (Table 1).

**Figure 4:**
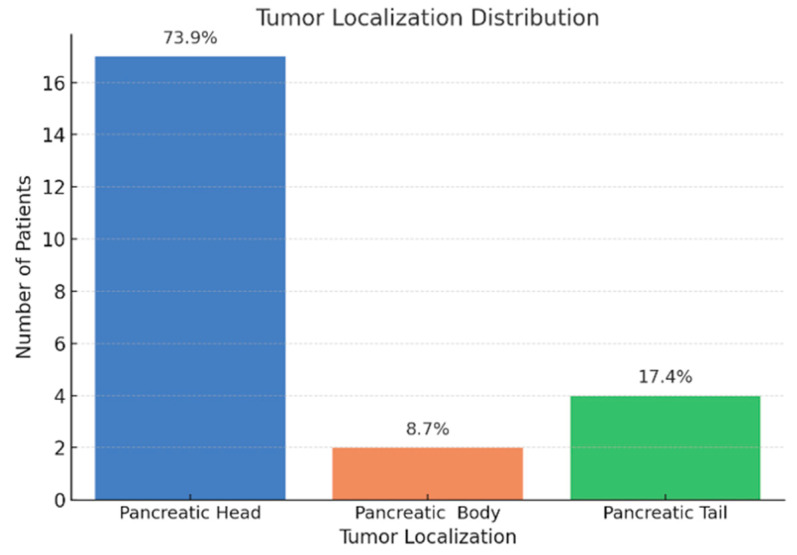
Anatomical distribution of pancreatic tumors in the study cohort (n = 23). Values are expressed as percentages of patients.

**Figure 5:**
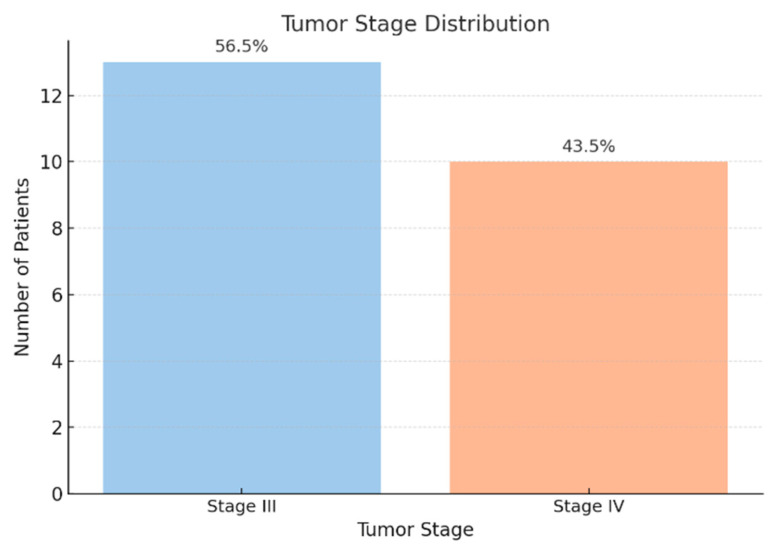
Tumor stage distribution among the study cohort (n = 23). Values are expressed as percentages of patients.

From a histological perspective, all tumors were identified as pancreatic ductal adenocarcinomas, with 73.9% located in the pancreatic head (Figure 4). In addition, all patients included in the study were diagnosed with advanced-stage disease (Figure 5).

### 3.3. Differential Expression and Validation of Circulating miRNAs

**Figure 6:**
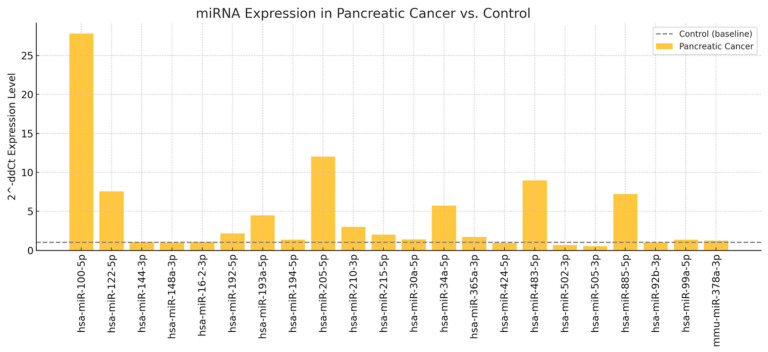
Screening phase: differential expression of circulating miRNAs in pooled plasma samples from patients with stage III–IV pancreatic cancer compared to healthy controls. Expression values were calculated using the comparative Ct method (2^−ΔΔCt^), with the healthy pooled sample as the calibrator (fold change = 1).

Subsequently, a screening step was performed for 176 miRNAs using two pooled plasma samples: one from the 23 patients with pancreatic cancer and one from 10 healthy controls, matched for age and sex distribution. Of the miRNAs analyzed, only 22 showed differential expression between patients with pancreatic cancer and healthy controls (Figure 6). The relative expression was calculated using the 2^−ΔΔCt^ method, where healthy controls served as the reference group (expression level normalized to 1) (Figure 6). With the aim of developing a diagnostic kit for patients with pancreatic cancer, we selected five miRNAs for validation in individual plasma samples: hsa-miR-100-5p (27.8-fold increase), hsa-miR-122-5p (7.5-fold), hsa-miR-885-5p (7.2-fold), hsa-miR-34a-5p (5.7-fold), and hsa-miR-193a-5p (4.4-fold) (Figure 6). This decision was based on a combination of criteria, including the magnitude of differential expression and prior evidence of involvement in pancreatic cancer pathogenesis. The remaining upregulated miRNAs that were not validated in this phase will be considered for follow-up studies.

**Figure 7:**
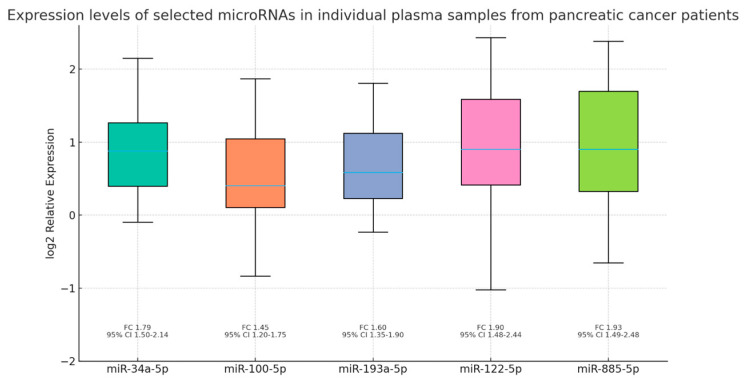
Validation of the five selected circulating miRNAs (miR-34a-5p, miR-100-5p, miR-193a-5p, miR-122-5p, and miR-885-5p) in individual plasma samples from pancreatic cancer patients compared to healthy controls. Boxplots represent the distribution of individual log2 relative expression values (median and interquartile range). Fold changes (FCs) and 95% confidence intervals (CIs) calculated from the mean log2 expression are shown below each plot. Expression values were calculated using the comparative Ct method (2^−ΔΔCt^), with the healthy pooled sample as the calibrator (fold change = 1). Group comparisons were performed using the Mann–Whitney U test, and statistical significance was confirmed after correction for multiple testing using the Benjamini–Hochberg false discovery rate (FDR). All five miRNAs remained significantly upregulated in pancreatic cancer plasma samples (unadjusted and FDR-adjusted *p* < 0.05).

In the second validation phase, we analyzed the expression levels of the five selected miRNAs—miR-34a-5p, miR-100-5p, miR-193a-5p, miR-122-5p, and miR-885-5p—in individual plasma samples. All five miRNAs were significantly upregulated in patients with pancreatic cancer compared to healthy controls (Mann–Whitney test, *p* < 0.0001), supporting their potential relevance as non-invasive biomarkers (Figure 7). Importantly, all results remained statistically significant after adjustment for multiple testing using the false discovery rate (FDR, Benjamini–Hochberg method; adjusted *p* < 0.05). Specifically, the significance levels were as follows: miR-34a-5p (*p* < 0.00001, FDR-adjusted *p* < 0.00001), miR-100-5p (*p* = 0.00005, FDR-adjusted *p* = 0.00005), miR-193a-5p (*p* < 0.00001, FDR-adjusted *p* < 0.00001), miR-122-5p (*p* < 0.00001, FDR-adjusted *p* < 0.00001), and miR-885-5p (*p* = 0.00005, FDR-adjusted *p* = 0.00005).

To further explore potential clinical or paraclinical parameters influencing the plasma expression of selected miRNAs, we performed a simple linear regression analysis. In this model, miRNA concentration served as the dependent variable, while tumor stage, smoking status, alcohol consumption, history of acute or chronic pancreatitis, and presence of diabetes mellitus were included as independent variables (Table 2). Among these, only tumor stage demonstrated a statistically significant positive association with the expression levels of miR-34a-5p, miR-100-5p, miR-193a-5p, miR-122-5p, and miR-885-5p (Table 2). Specifically, regression coefficients and 95% confidence intervals confirmed significance for all five miRNAs (*p* < 0.05), while the strength of association, expressed by Pearson correlation coefficients, ranged from r = 0.186 to r = 0.398, corresponding to R^2^ values between 0.034 and 0.158. Notably, miR-100-5p exhibited the strongest correlation with tumor stage (r = 0.398, R^2^ = 0.158), suggesting a moderate effect size, whereas the other four miRNAs showed weaker, though still significant, associations. By contrast, smoking, alcohol consumption, diabetes mellitus, and pancreatitis were not significantly associated with circulating miRNA levels in this analysis. However, given the limited sample size of our pilot cohort, these negative findings should be interpreted with caution, and larger multicenter studies will be necessary to clarify their potential impact.

## 4. Discussion

### 4.1. Patient Characteristics

The female predominance observed in our cohort of pancreatic cancer patients does not align with data from the current literature, which generally reports a higher risk of this malignancy among men [12,13]. However, recent studies have highlighted a rising incidence of pancreatic cancer in both sexes [14]. We acknowledge that the disproportionate representation of women in our study is most likely related to sampling bias inherent to the relatively small, single-center cohort, which may limit the generalizability of our findings. Regarding age distribution, the majority of patients were within the 60–69-year age group (40.9%), followed by those aged 50–59 years (27.3%) and 70–79 years (18.2%). These results are consistent with the existing literature, which indicates an increased risk of pancreatic cancer with advancing age [14].

Regarding the comorbidities observed among the patients included in our study, we noted a high prevalence of smoking and alcohol consumption, as well as diabetes mellitus and gallstone disease. Both alcohol use and smoking are among the most important risk factors for pancreatic cancer, while diabetes mellitus may act as both a risk factor and a consequence of this malignancy [15]. The unexpectedly high prevalence of gallstone disease (34.8%) may be partially explained by the predominance of female sex in our study cohort. Additionally, a considerable proportion of patients (26.1%) reported a history of Helicobacter pylori infection. Emerging evidence suggests the potential involvement of H. pylori in the pathogenesis of both autoimmune pancreatitis and pancreatic ductal adenocarcinoma [16,17]. In H. pylori-positive individuals, the reduction in antral D cells leads to a decrease in somatostatin secretion, which in turn results in a compensatory increase in secretin release [17]. Secretin has been shown to promote pancreatic tissue growth and may thereby contribute to carcinogenesis [17]. Furthermore, H. pylori is considered a contributor to microbiome dysbiosis—referred to as the “oncobiome”—which may promote abnormal cellular proliferation and tumor development [17].

All patients included in our study were diagnosed with stage III or IV disease according to the TNM classification system, and the majority of tumors (73.9%) were located in the pancreatic head. It is now well established that the poor prognosis of pancreatic cancer is primarily due to its late diagnosis, which frequently occurs at an advanced stage [18,19]. The predominance of cephalic pancreatic tumors is consistent with the frequent presence of cholestatic syndrome, which can be explained by tumor-induced compression of the common bile duct. Furthermore, 43.5% of patients were diagnosed with stage IV disease, all of whom exhibited hepatic metastases. Consequently, both the cholestatic and hepatocellular injury syndromes observed in this cohort may also be attributed to the presence of secondary hepatic tumors.

### 4.2. Validation of Circulating miRNAs

During the screening phase of our study, we analyzed miRNA profiles in two pooled plasma samples: one obtained from patients with stage III–IV pancreatic cancer and the other from healthy control subjects, using real-time PCR. Out of the 176 miRNAs initially investigated, only 22 exhibited differential expression in patients with pancreatic cancer. These included: miR-100-5p, miR-122-5p, miR-144-3p, miR-148a-3p, miR-16-2-3p, miR-192-5p, miR-193a-5p, miR-194-5p, miR-205-5p, miR-210-3p, miR-215-5p, miR-30a-5p, miR-34a-5p, miR-365a-3p, miR-424-5p, miR-483-5p, miR-502-3p, miR-505-3p, miR-885-5p, miR-92b-3p, miR-99a-5p, and miR-378a-3p. In the second phase, we validated in individual plasma samples the overexpression of the five miRNAs that exhibited the highest fold changes during screening in pancreatic cancer patients: miR-34a-5p, miR-100-5p, miR-193a-5p, miR-122-5p, and miR-885-5p. Moreover, we observed a statistically significant correlation between the plasma levels of these biomarkers and tumor stage, suggesting their potential prognostic relevance. These results provide preliminary evidence that specific circulating miRNAs may distinguish patients with advanced pancreatic cancer from healthy controls and reflect disease stage. Although not yet applicable to routine clinical practice, our findings add to the growing body of evidence supporting the role of circulating miRNAs in pancreatic cancer biology and justify further investigation in larger, prospectively designed cohorts.

### 4.3. Comparison with Literature

Ding et al. recently investigated the pathophysiological mechanisms through which exosomes secreted by human umbilical-cord-derived mesenchymal stem cells (hucMSC) contribute to the development of pancreatic ductal adenocarcinoma (PDAC) [20]. The oncogenic effects of hucMSCs are attributed to these exosomes, which deliver specific proteins and miRNAs to cancer cells [20]. The authors used mice subcutaneously injected with Panc-1 cells. In a subset of these animals, hucMSC-derived exosomes were injected intratumorally. They observed a significantly faster tumor growth in mice receiving the exosome injection compared to those injected with Panc-1 cells alone [20]. Furthermore, a significant upregulation of miR-100-5p, as well as miR-148a-3p, miR-143-3p, miR-21-5p, and miR-92-3p, was identified in the groups treated with hucMSC exosomes [20]. Based on these findings, the authors hypothesized that hucMSC-derived exosomes may promote pancreatic tumor cell proliferation through the transfer of overexpressed miRNAs [20]. Our study supports these results by identifying elevated expression not only of miR-100-5p, but also of miR-148a-3p and miR-92-3p in patients with pancreatic cancer compared to control subjects.

Another study employing pancreatic cancer cell lines (BxPC-3, Panc-1, S2-007) aimed to identify novel miRNAs involved in the progression of PDAC through the transforming growth factor beta (TGF-β) signaling pathway [21]. The authors demonstrated that TGF-β induces the overexpression of key epithelial-to-mesenchymal transition (EMT) transcription factors such as Snail family transcriptional repressor 1 (SNAI1), Snail family transcriptional repressor 2 (SNAI2) and cadherine 2 (CDH2), while downregulating cadherine 1 (CDH1), an important intercellular adhesion molecule (*p* < 0.05). Additionally, they confirmed the transcriptional induction of miR-100 and miR-125b by TGF-β [21]. Accordingly, our finding of miR-100-5p overexpression in patients with pancreatic cancer is strongly supported by evidence linking this micRNA to pancreatic oncogenesis. Moreover, by identifying a positive correlation between miR-100-5p expression levels and tumor stage, we further support the hypothesis that this miRNA contributes to the metastatic progression of pancreatic tumor cells.

The data available in the literature regarding the role of miR-122-5p in pancreatic oncogenesis are contradictory. In a recently published article, Mazza et al. reported *miR-122-5p* as an independent negative prognostic factor in patients with pancreatic cancer (*p* = 0.037) [22]. Specifically, plasma levels of miR-122-5p and miR-6126 were significantly higher in patients with metastatic pancreatic cancer compared to those without metastases [22]. Moreover, miR-122-5p levels were correlated with tumor stage [22]. Given the stage-dependent expression of miR-122-5p in our cohort (*p* = 0.01; 95% CI 3.49–4.21), our results are consistent with those of Mazza et al., supporting the role of miR-122-5p as a negative prognostic biomarker in pancreatic cancer. In contrast to the findings discussed above, Dai et al. reported a tumor-suppressive role for miR-122-5p, demonstrating its involvement in the inhibition of pancreatic cancer cell proliferation, migration, and invasion [23]. Using real-time PCR, the authors showed that miR-122-5p was significantly downregulated in pancreatic tumor tissue compared to normal pancreatic tissue [23]. This study supports the hypothesis that miR-122-5p overexpression may inhibit pancreatic cancer cell proliferation by inducing cell cycle arrest in the G0/G1 phase and increasing the rate of tumor cell apoptosis [23]. These apparently conflicting results may be explained by the different biological contexts in which miR-122-5p was evaluated. Plasma miRNA levels reflect not only tumor-derived secretion but also release from stromal, inflammatory, or hepatic cells, whereas tissue-based expression directly reflects tumor cell activity. Another possible confounding factor is cholestasis or hepatocellular injury, frequently encountered in patients with advanced pancreatic cancer. Additional discrepancies could also arise from methodological and pre-analytical variability, including sample type (plasma vs. tissue), normalization strategies, and the heterogeneity of patient cohorts.

Ganepola et al. confirm the utility of a panel consisting of three miRNAs—miR-885-5p, miR-22, and miR-642b—for the early diagnosis of pancreatic cancer [24]. The sensitivity and specificity rates of this panel reached 91%, compared to the sensitivity rate of CA19-9, which did not exceed 73% [24]. In contrast to the aforementioned findings, our study demonstrated not only the overexpression of miR-885-5p in patients with pancreatic cancer but also a positive correlation between this miRNA and tumor stage. Further studies on larger patient cohorts are therefore warranted to assess miR-885-5p expression in pancreatic cancer and to elucidate the mechanisms through which it may be involved in tumorigenesis.

MiR-34a-5p was another miRNA found to be overexpressed in patients with pancreatic cancer included in our study, compared to control subjects. This finding does not align with existing data from the literature [25,26,27]. For instance, Hidalgo-Sastre et al. recently reported the role of miR-34a in limiting the progression of pancreatic ductal adenocarcinoma by modulating the tumor microenvironment [25]. These authors demonstrated that reduced expression of miR-34a was associated with increased levels of proinflammatory cytokines, particularly in pancreatic acinar cells [25]. This, in turn, led to activation of the Nuclear Factor kappa-light-chain-enhancer of activated B cells (NF-κB) and Phosphorylated Signal Transducer and Activator of Transcription 3 (*p*-STAT3) pathways—both of which promote the development of preneoplastic lesions and carcinogenesis—along with the recruitment of additional inflammatory cells, ultimately accelerating the progression of pancreatic ductal adenocarcinoma [25]. One possible explanation for this finding is that most research studies have assessed miR-34 expression in tumor tissue samples or pancreatic cancer cell lines rather than in plasma. Circulating miRNAs, however, may derive not only from tumor cells but also from stromal and immune cells within the tumor microenvironment, or from distant tissues responding to systemic inflammation and tumor burden. Consequently, plasma overexpression of miR-34a-5p could reflect a compensatory host response or release from apoptotic/necrotic tumor cells, whereas reduced tumor tissue levels may represent loss of tumor-suppressive function within malignant cells. In addition, methodological differences such as sample type (tissue vs. plasma), normalization strategies, and patient cohort heterogeneity could further contribute to the divergent findings reported across studies. Taken together, these observations suggest that the role of miR-34a-5p in pancreatic cancer is context-dependent, underscoring the importance of parallel assessment in both tissue and blood compartments in future studies.

Our study identified an increased expression of miR-193a-5p in patients with pancreatic cancer compared to control subjects. This finding is supported by existing literature highlighting the role of miR-193a-5p in pancreatic cancer progression and metastasis [28,29]. Li et al. demonstrated a direct correlation between miR-193a-5p expression levels, lymph node metastasis, and overall survival rates in these patients [28]. Moreover, their study revealed the pro-invasive effects of miR-193a-5p, mediated by the downregulation of SRSF6 [28]. By confirming a positive association between miR-193a-5p expression and tumor stage in our cohort (*p* = 0.004, 95% CI 3.49–4.17), our results further support these findings.

To date, no clinically validated or Food and Drug Administration/European Medicines Agency (FDA/EMA)-approved diagnostic or prognostic kit based on circulating miRNAs exists for pancreatic cancer. Although several panels of miRNAs, including some of those evaluated in our study, have been repeatedly reported in systematic reviews and meta-analyses as promising biomarkers, they remain at an exploratory stage and require validation in larger, multicenter cohorts before clinical implementation [30,31].

### 4.4. Limitations and Future Directions

An important limitation of our study is the relatively small sample size (23 patients and 10 controls), which reduces statistical power and limits the generalizability of our findings. This pilot study was designed primarily to generate exploratory hypotheses and to identify circulating miRNAs of potential interest for future validation. Another limitation is that only patients with advanced-stage disease (stage III–IV) were included, which restricts the conclusions that can be drawn regarding the diagnostic performance of circulating miRNAs in early-stage pancreatic cancer. Future studies should therefore include patients with earlier stages of disease as well as disease control groups (e.g., chronic pancreatitis or benign biliary obstruction) to better assess specificity and clinical utility. Accordingly, we recognize that robust biomarker validation will require larger, multicenter cohorts, ideally including early-stage cases and appropriate disease controls, to establish the clinical applicability of these results.

While our findings highlight a distinct circulating miRNA signature associated with advanced pancreatic cancer, their integration into a diagnostic or prognostic kit remains a long-term goal. At this stage, the results should be regarded as exploratory, requiring confirmation in larger, multicenter cohorts. Future research should focus on validating these biomarkers in early-stage pancreatic cancer and in disease control groups, as well as on assessing their incremental value when combined with established markers such as CA19-9. Only after rigorous validation across diverse populations could these miRNAs be realistically considered for incorporation into clinically applicable diagnostic or prognostic tools.

## 5. Conclusions

This study identified a distinct circulating microRNA signature associated with advanced pancreatic ductal adenocarcinoma. Among 176 miRNAs screened in pooled plasma samples, 22 were found to have differential expression in patients with pancreatic cancer compared to age- and sex-matched controls. Subsequent validation in individual plasma samples confirmed the significant overexpression of five miRNAs—miR-34a-5p, miR-100-5p, miR-193a-5p, miR-122-5p, and miR-885-5p—all of which demonstrated a positive correlation with tumor stage, supporting their potential prognostic relevance. These findings highlight the feasibility of using circulating miRNAs as minimally invasive biomarkers for disease detection and staging in pancreatic cancer. However, further large-scale studies are warranted to validate these results and to determine the utility of these biomarkers in early-stage disease or in high-risk populations with premalignant pancreatic lesions. Integrating circulating miRNA profiling into clinical practice may enhance diagnostic capabilities and contribute to improved patient stratification and personalized treatment strategies.

## Figures and Tables

**Table 1:** Descriptive statistics of hematological, biochemical and inflammatory markers in the study cohort (n = 23). Abbreviations: std—standard deviation; min—minimum value; max—maximum value; 25%, 50%, 75%—percentiles representing the first quartile, median, and third quartile, respectively.

Biological Marker	Mean	Std	Min	25%	50%	75%	Max
Hemoglobin	12.11	1.53	8.5	11.25	12.22	13.1	14.7
White blood cells	7985.65	3119.04	3720.0	5455.0	7010.0	9605.0	15,300.0
Platelets	263,695.65	90,904.13	89,800.0	205,800.0	242,000.0	317,500.0	428,000.0
Glucose	118.7	35.03	78.0	101.0	115.0	126.5	247.0
Aspartate aminotransferase	218.13	225.36	17.0	35.5	156.0	256.0	724.0
Alanine aminotransferase	284.22	333.0	15.0	53.0	125.0	467.5	1039.0
Amylase	115.33	125.01	10.6	43.5	52.0	153.5	519.0
Lipase	437.8	684.96	7.0	61.2	88.5	515.75	2811.0
Total Bilirubin	10.39	12.16	0.43	1.12	4.2	18.12	40.29
Direct Bilirubin	7.97	9.78	0.19	0.44	3.08	14.34	29.0
Gamma-glutamyl transferase	674.04	861.81	10.0	131.0	256.0	849.0	3086.0
Alkaline phosphatase	426.49	336.99	50.28	183.5	324.0	610.5	1225.0
Cholesterol	188.3	60.06	120.0	136.4	183.0	223.5	355.0
Triglycerides	166.61	102.03	67.0	112.5	133.0	171.5	485.0
C-reactive protein	30.67	13.63	0.93	23.0	32.0	40.06	56.0
Erythrocyte sedimentation rate	35.8	17.27	0.4	30.5	34.0	42.5	70.0
Uric acid	5.59	1.1	3.0	4.8	5.7	6.35	7.2

**Table 2:** Simple linear regression analysis of clinical factors influencing plasma expression levels of selected miRNAs in pancreatic cancer patients.

Predictor	miR-34a-5p 95% CI ^1^ *p* Value Pearson r R^2^	miR-100-5p 95% CI ^1^ *p* Value Pearson r R^2^	miR-193a-5p 95% CI ^1^ *p* Value Pearson r R^2^	miR-122-5p 95% CI ^1^ *p* Value Pearson r R^2^	miR-885-5p 95% CI ^1^ *p* Value Pearson r R^2^
Tumor stage	3.44–4.22 0.04 0.213 0.045	3.52–4.08 0.004 0.398 0.158	3.49–4.17 0.01 0.238 0.057	3.49–4.21 0.01 0.186 0.034	3.47–4.16 0.02 0.202 0.041
Age	0.03–0.01 0.40 −0.183 0.033	0.03–0.02 0.55 −0.130 0.017	0.03–0.01 0.56 −0.126 0.016	0.05–0.02 0.44 −0.166 0.028	0.04–0.02 0.56 −0.126 0.016
Gender	0.79–0.40 0.50 −0.146 0.021	0.91–0.34 0.35 −0.202 0.041	0.77–0.35 0.44 −0.167 0.028	0.89–0.81 0.92 −0.021 0.000	0.93–0.80 0.87 −0.036 0.001
Smoking	0.91–1.77 0.53 −0.203 0.041	0.97–1.64 0.22 −0.387 0.150	0.94–1.73 0.45 −0.248 0.062	0.95–1.78 0.62 −0.150 0.022	0.94–1.72 0.44 −0.249 0.062
Alcohol consumption	1.20–1.99 0.53 −0.064 0.004	1.35–1.99 0.81 −0.081 0.007	1.28–2.02 0.74 −0.036 0.001	1.19–1.94 0.37 −0.087 0.008	1.22–1.95 0.42 −0.124 0.015
Diabetes mellitus	1.33–2.17 0.36 0.085 0.007	1.34–2.02 0.47 −0.041 0.002	1.31–2.09 0.47 0.019 0.000	1.35–2.15 0.33 −0.029 0.001	1.32–2.10 0.44 −0.002 0.000
Acute/Chronic pancreatitis	1.60–2.22 0.60 0.137 0.019	1.63–2.13 0.67 0.115 0.013	1.65–2.21 0.44 0.176 0.031	1.68–2.26 0.29 0.230 0.053	1.62–2.20 0.49 0.161 0.026

^1^ CI = Confidence interval.

## Data Availability

The data that support the findings of this study are available from the corresponding author upon reasonable request.
